# Correlations between patient-perceived outcome and objectively-measured biomechanical change following Total Knee Replacement

**DOI:** 10.1016/j.gaitpost.2019.02.028

**Published:** 2019-05

**Authors:** P.R. Biggs, G.M. Whatling, C. Wilson, C.A. Holt

**Affiliations:** a Cardiff School of Engineering, College of Physical Sciences, Cardiff University, Cardiff, United Kingdom; b Arthritis Research UK Biomechanics and Bioengineering Centre, Cardiff University, Cardiff, United Kingdom; c Department of Trauma and Orthopaedics, University Hospital of Wales, Cardiff, United Kingdom

**Keywords:** Gait analysis, Biomechanics, Total Knee Replacement, Knee osteoarthritis, Principal Component Analysis, Patient reported outcome measures

## Abstract

**Background:**

Total Knee Replacement (TKR) surgery is being utilised in a younger, more active population with greater functional expectations. Understanding whether patient-perceived measures of function reflect objective biomechanical measures is critical in understanding whether functional limitations can be adequately captured within a clinical setting.

**Research Question:**

Do changes in objective gait biomechanics measures reflect patient-reported outcome measures at approximately 12 months following TKR surgery?

**Methods:**

Three-dimensional gait analysis was performed on 41 patients with OA who were scheduled for TKR surgery, 22 of which have returned for a (9–24 month) follow-up assessment. Principal Component Analysis was used to define features of variation between OA subjects and an additional 31 non-pathological control subjects. These were used to train the Cardiff Classifier, an objective classification technique, and subsequently quantify changes following TKR surgery. Patient-perceived changes were also assessed using the Oxford Knee Score (OKS), Knee Outcome Survey (KOS), and Pain Audit Collection System scores (PACS). Pearson and Spearman correlation coefficients were calculated to establish the relationship between changes in objectively-measured and perceived outcome.

**Results:**

Objective measures of biomechanical change were strongly correlated to changes in OKS(r=-0.695, p < 0.001) and KOS(r=-.810, p < 0.001) assessed outcomes. Pain (PACS) was only related to biomechanical function post-operatively (r=-.623, p = 0.003).

**Significance:**

In this biomechanics study, the relationship between changes in objective function and patient-reported measures pre to post TKR surgery is stronger than in studies which did not include biomechanics metrics. Quality of movement may hold more significance for a patient’s perception of improvement than functional measures which consider only the time taken or distance travelled during functional activities.

## Highlights

• Osteoarthritic gait was accurately classified using Dempster Shafer Theory.
• Biomechanical deficits exist following TKR surgery.
• Objective biomechanical improvement strongly correlated with subjective outcome measures.
• Pre-operative pain did not correlate with gait biomechanics.

## 1 Introduction

The principal goal of Total Knee Replacement (TKR) in the treatment of knee osteoarthritis (OA) is to improve quality of life through the restoration of joint function, and reduction of pain. In recent years, there has been a dramatic rise in the utilisation of TKR to treat younger patients [1], and those with higher functional expectations [2,3]. Changes in physical function following surgery have most commonly been monitored using patient-reported outcome measures (PROMs). Recent evidence suggests PROMs fail to capture changes in performance-based measures following TKR surgery [[4], [5], [6], [7]]. It has also been suggested that patients with severe OA have difficulty discriminating between functional limitation and pain when self-assessing their ability to perform activities of daily living [4].

Gait analysis provides an objective approach for assessing the apparent disparity between performance-based and perceived functional changes pre to post TKR surgery. Numerous studies have reported functional deficits in biomechanical parameters in TKR cohorts when compared to healthy subjects [8]. Few studies, however, have discussed whether patients with the greatest perceived recovery also have the best biomechanical outcomes and vice versa.

Biomechanical gait analysis yields a wealth of information regarding joint kinematics and kinetics, but the interpretation of findings is complicated by interdependencies of the biomechanical variables [9]. As a consequence there has been growing interest in statistical techniques which objectively summarise pathological gait changes relative to normative population [[9], [10], [11]]. One of the challenges to summarising biomechanical data is the reduction of temporal waveforms into discrete metrics. One popular method is Principal Component Analysis (PCA), which reduces data into orthogonal components of variation. This method is objective, therefore requiring no subjective selection of target features such as waveforms peaks, and has been demonstrated in numerous studies to successfully identify subtle differences between movement patterns [12,13].

In our unit, the application of PCA has been combined with a classification method based on a Dempster-Shafer theory of evidence, termed the ‘Cardiff Classifier’. This method has been demonstrated to accurately characterise the biomechanical changes in late-stage OA subjects [14] as a basis for measuring recovery following subsequent TKR [[15], [16], [17]]. Applying these techniques to lower-limb biomechanics during level gait, disparities between the magnitude of subjective and objective functional recovery have been highlighted [16]. Realistic goals following surgery may, however, differ between objective and subjective outcome measures. For example, the Oxford Knee Score (OKS) is designed specifically to be responsive to perceived changes following TKR surgery, meaning healthy subjects would fall into a narrow band within the outcome measure, generally achieving a perfect score of 0/48. Objective methods such as knee range of motion or gait classification, however, are not designed specifically to be responsive to changes following TKR, hence healthy subjects generally fall within a larger portion of the outcome measure.

This study aims to assess the relationship between patient-perceived outcome and objective biomechanical classification of level gait. The first objective is to compare the level of change in PROMs and of biomechanical classification of level gait following TKR surgery. The second objective is to address whether the assessment of functional gains following surgery is significantly altered using gait classification in comparison to using PROMs alone.

## 2 Methods

### 2.1 Study participants

The study was approved by the Research Ethics Committee for Wales and Cardiff and Vale University Health Board. Forty-one patients with knee OA who were listed for primary TKR surgery at Cardiff and Vale Orthopaedic Centre were recruited into the study. Subjects were excluded if they were unable to walk 10 m without a walking aid, were unable to give informed consent, had rheumatoid arthritis, or had an unrelated musculoskeletal, neurological or visual condition which might affect the way they move. Participants with bilateral OA were not excluded, nor were those whom had undergone previous arthroplasty in other lower limb joints. At the time of analysis, 22 subjects had undergone re-assessment at least 9 months post-operatively. Due to several practical issues, there was variability in the timing of follow-up visit – the median time was 13.2 months however this ranged between 9.3 and 22.8 months following surgery.

Thirty-one volunteers with no lower-limb pathology (NP) were also recruited from University staff, students and the wider community using poster and email advertisements. Subjects were excluded if they had a history of a lower-limb musculoskeletal condition which required medical treatment, had self-reported pain in the lower-limb or back, or had an inflammatory, neurological or visual condition which might affect the way they move.

### 2.2 Biomechanical analysis

Human motion analysis was performed during level gait at the motion analysis laboratory at Cardiff School of Engineering. A lower-limb CAST marker set [18] was attached to subjects, while they walked barefoot at a self-selected pace along a 10 m walkway. Marker trajectories were collected using 8 Oqus (Qualisys, Sweden) cameras capturing at 60 Hz, and ground reaction forces were calculated from two force platforms (Bertec, USA) capturing at 1080 Hz. Hip, knee and ankle kinematics and kinetics were calculated within Visual 3D (C-Motion, USA).

### 2.3 Patient-reported outcome

Two validated outcome measures which assess perceived pain and function; the OKS and the Knee Outcome Survey (KOS), were collected at pre and post-operative visits. A post-operative KOS score was missing for one subject. During the study, the Pain Audit Collection System (PACS) was added as an additional pain-centred outcome measure following approval of a suitable ethical amendment. Information on PACS is therefore only complete for 16/22 patients at baseline, and 20 post-operatively. A PACS score was missing in error for one subject, resulting in n = 15 for pre-post PACS comparisons.

### 2.4 Data reduction

PCA was performed on the waveforms of OA and NP subjects to define distinct biomechanical features of variation between and within the cohorts. The first three principal components were initially selected for each input variable, resulting in 72 discrete variables per subject. The Cardiff Classifier was then used to rank input variable importance. This ranking deviated from a previously reported method [17], and to reduce the risk of over-fitting the training data was split into two halves and the Cardiff Classifier was used to rank the input variables within both data sets. There were 18 biomechanical variables which were identified as being highly ranked in each group and were retained for further analysis.

### 2.5 Data classification

The 18 discrete biomechanical features of 31 NP and 41 OA subjects were used to train the Cardiff Classifier on the characteristics of OA gait. This process defines the relationship between each of the input features, and a belief value of OA, NP and Uncertainty. These three belief values termed B(OA), B(NP) and U respectively are then used to classify between OA and NP gait biomechanics [15]. If, for example, B(OA) is greater than B(NP), and the subject belongs to the OA group, the classification technique is deemed to have successfully classified this subject. The robustness of this classification was assessed using the leave-one-out (LOO) cross-validation algorithm [19]. Using LOO, the classification control parameters are defined using *n-1* subjects, and the belief values are then calculated for the remaining subject. This technique is repeated until every subject has been classified.

The same process was then applied to the lower-limb biomechanics collected at the follow-up visit. The previously defined principal components were used to calculate scores for the 22 subjects following surgery. The same 18 biomechanical features were then used in the trained classifier to calculate the three belief values B(OA), B(NP) and B(U) at the follow-up visit. The objective change in gait biomechanics was defined as the difference between the pre and post-operative B(OA).

### 2.6 Statistical analysis

All statistical inferences were calculated using SPSS (IBM, USA). Linear correlations between B(OA) and PROMs were assessed using the Pearson’s correlation coefficient (parametric data) or Spearman’s Rank Correlation Coefficient (nonparametric data). The effect size was calculated as the mean difference divided by the pooled standard deviation.

## 3 Results

**Fig. 1:**
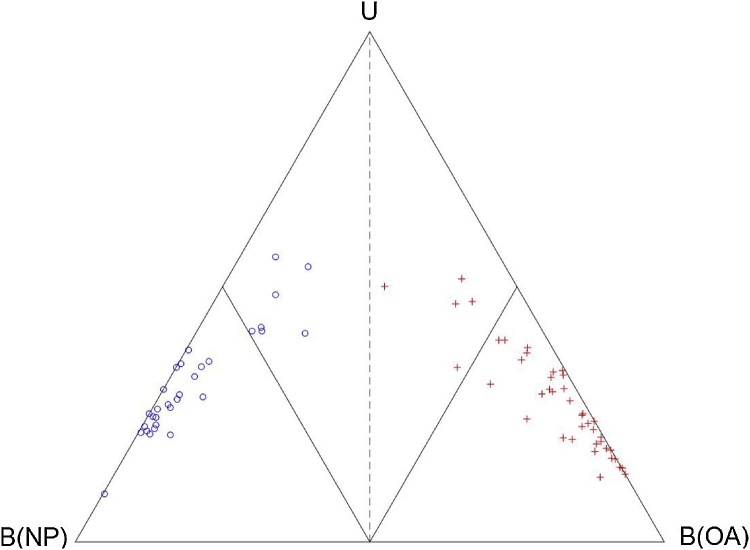
Simplex plot of the classification of the 31 NP (blue circle) and 41 OA (red cross) subjects which were used to train the Cardiff Classifier on the biomechanical features of severe osteoarthritic gait. The three vertices represent the points where B(NP), B(OA) and B(U) = 1 (100%). The decision boundary where B(OA)=B(NP) is shown as a dashed line. The boundaries where B(OA) = 0.5 and B(NP) = 0.5 are shown as interior solid lines. (For interpretation of the references to colour in this figure legend, the reader is referred to the web version of this article).

The demographics of participants used to train the Cardiff Classifier are shown in Table 1. The OA cohort had significantly higher BMI and body mass and were significantly older than the NP cohort. The Cardiff Classifier was able to correctly classify between NP and OA gait biomechanics in all 72 cases, assessed using the LOO cross-validation technique. The three belief values are shown in a simplex plot within Fig. 1.

**Table 1:** Average demographics of the 31 non-pathological (NP) and 41 late-stage osteoarthritic (OA) subjects which were used to train the Cardiff Classifier. Standard deviation is shown in brackets. *Significant differences (p < 0.05) between OA and NP cohort.

	Number	BMI	Height/m	Mass/kg	Age/years	Gender
NP	31	24.6 (4.0)	1.69 (0.09)	70.32 (14.5)	40.7 (17.9)	12 M 19F
OA	41	32.5 (6.4)*	1.67 (0.10)	91.28 (20.3)*	68.4 (8.6)*	19 M 22F

The pre-post changes in the objective and subjective outcome measures for the 22 subjects who returned post-operatively are displayed in Table 2. There were statistically significant increases (improvements) in all PROMs, alongside expected increases in B(NP) and reduction in B(OA). The effect size of all these changes was large (>0.8) [20], however, the subjective outcome scores appear to be a more responsive measure of changes following TKR. There was also a significant moderate increase in uncertainty within the classification following surgery.

**Table 2:** Changes in the objective and subjective measures of function between baseline (pre-operative) and post-operative (9+ month post-operative) visit. The objective belief values: belief of OA function, B(OA), belief in non-pathological function, B(NP), and uncertainty U, were calculated using the Cardiff Classifier. Subjective PROMs were calculated as a percentage of the total score, where 100% indicates heathy function.

		Objective			Subjective		
		B(OA)	B(NP)	U	OKS (%)	KOS (%)	PACS (%)
Baseline	Mean	0.662	0.046	0.292	42.8	46.1	53.5
	STD	0.152	0.051	0.113	21.8	20.1	23.7
	n	22	22	22	22	22	16
Post-operative	Mean	0.511	0.141	0.348	73.3	73.7	83.4
	STD	0.204	0.14	0.113	18.9	23.0	21.1
	n	22	22	22	22	21	20
Change	Diff	−0.151	0.095	0.056	30.5	27.6	29.9
	*p*	<0.001	.001	<0.001	<0.001	<0.001	<0.001
	Effect size	−0.848	0.990	0.501	1.36	1.42	1.34

Correlations between the objectively measured gait function, B(OA), and the various PROMs are shown in Table 3. Moderate to strong correlations existed between B(OA) and two of the PROMS, OKS and KOS, both before and after surgery. This relationship appears even stronger if considering the magnitude of change in these measures following surgery. There was, therefore, a good level of agreement between these measures in terms of which subjects improved the most and least following surgery.

**Table 3:** Correlations between the Cardiff Classifier belief B(OA) and each of the patient-reported outcomes at baseline (pre-operative), post-operatively, and the post-surgical change for the 22 TKR subjects. * P < 0.05, *P < 0.01, ^†^ Spearman’s correlation coefficient (non-parametric data).

			KOS	OKS	PACS
B(OA)	Baseline	r	−.695^**†^	−.621^**^	−.261
		p	.000	.002	.328
		N	22	22	16
	Postoperative	r	−.656^**†^	−.685^**†^	−.623^**†^
		p	.001	.000	.003
		N	21	22	20
	Change	r	−.741^**^	−.810^**^	−.234
		p	.000	.000	.402
		N	21	22	15

The relationship between PACS and B(OA) was only present following TKR surgery. Changes in biomechanical function following surgery were therefore not linearly correlated to reductions in pain assessed using the PACS outcome measure.

**Fig. 2:**
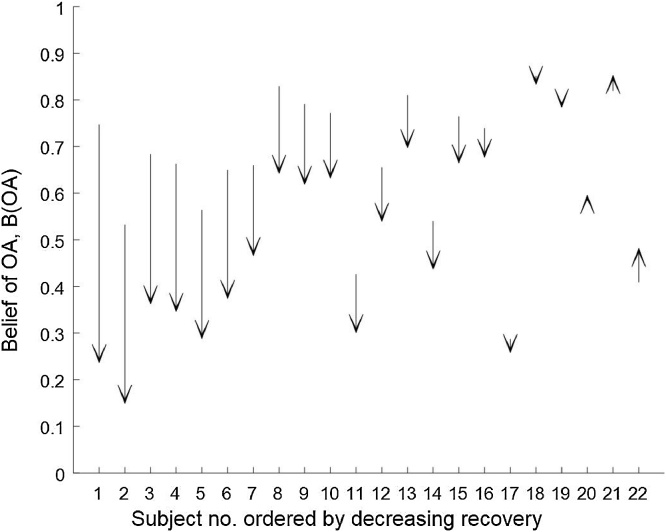
Arrow plot of change in B(OA) for each subject following TKR surgery. Subjects have been ordered in decreasing functional recovery, where small worsening of biomechanical function is observed in subjects.20–22.

Fig. 2 shows an arrow plot of changes in B(OA) for each of the 22 subjects following TKR. The subjects have been ranked in order of improvement such that a relationship between pre-operative status and post-operative recovery can be visually assessed. There was no evident relationship between pre-operative B(OA), and a reduction in B(OA) following surgery.

## 4 Discussion

### 4.1 Gait biomechanics and perceived functional status

The key finding in this study is that restoration of gait biomechanics after surgery was strongly correlated with changes in OKS and KOS scores. The strength of the relationship in this cohort was surprising as these PROMs assess a much broader range of disability and function, if considering the International Classification of Functioning, Disability and Health [21], than that of a biomechanical assessment of level gait. Since an accepted target of satisfactory recovery of gait biomechanics following TKR has yet to be established, interpretation of this finding requires careful consideration. It cannot be inferred that good patient-perceived recovery results in good biomechanical recovery or vice versa. A particularly strong relationship (r = 0.81, p < 0.001) between change in OKS score and change in B(OA) does, however, indicate that interpretation of who improved the most and least following surgery would have been similar using the two measures.

The results of this study are in contrast with previous evidence that PROMs do not adequately capture performance-related changes following surgery [[4], [5], [6], [7]]. There are, however, important methodological differences in the objective performance-related measures considered within these studies. Comparisons between perception-based and performance-based measures in TKR cohorts have typically compared common PROMs with maximal performance tests which offer no information on quality of movement, such as the Timed Up and Go [4,5,22], Six Minute Walk [4,5,23], Stair Test [4,5], and Five Sit to Stand [7] tests. In contrast, objective quantification of the B(OA) using a combination of PCA and the Cardiff Classifier uses *only* measures which indicate the quality of movement. In this context, the quality of movement is determined by an objective definition of whether the kinematic or kinetic waveform is characteristic of severe OA, or of NP gait. As objective biomechanical measures arguably assess a smaller domain of disability and function than the aforementioned clinical performance tests, it is surprising that other studies have demonstrated only moderate correlations with PROMs. It is possible that the patient’s perception of functional improvement is more related to measures of quality of movement than those of time taken or distance travelled, which may be sensitive to aerobic capacity, comorbidities, and may be dominated by pain [24]. Future studies should assess whether the objective biomechanical change is reflected within, or associated with, changes in clinical performance-based measures, such as those recommended by the Osteoarthritis Research Society International [25].

There are far fewer biomechanical studies with a pre-post intervention design which compare biomechanical gait parameters and patient-perceived changes following TKR. In a cohort of 24 patients, Senden et al. [24] identified only a few, weak (r = 0.23-0.41) correlations between acceleration-based gait parameters and PROMs. In a cohort of 21, Liebensteiner et al. [26] demonstrated a relationship between PROMs and gait analysis was only found in one in eight of the tested gait parameters following surgery. Neither of these studies tested the relationship between post-operative change in these measures, and no summary measure was considered. Naili et al. [27] recently reported no significant correlations between the Gait Deviation Index (GDI), a summary measure considering only kinematics, and PROMs or performance-based measures. Naili et al. did, however, observe low-moderate correlations between the GDI-Kinetic, the equivalent measure using only kinetic information, and two subscales of the Knee Injury and Osteoarthritis Outcome survey.

The current study differs from these biomechanical studies in that the primary hypothesis being tested is that *changes* in objective measures correlate with *changes* in subjective measures. The summary gait measure considered within this study, a combination of PCA and the Cardiff Classifier, is also unique and has potential methodological advantages over the GDI. The biomechanical features of variance have been described using healthy and OA gait, as opposed to multiple gait pathologies, and hence may be more sensitive to changes resulting from OA. Gait kinematics and kinetics (including ground reaction forces) are also included in a single measure, and all frontal and transverse plane measures at the hip, knee and ankle were considered. In fact, three of the five most discriminatory biomechanical features were within variables not considered in either the GDI or GDI-kinetic. These differences might explain the much stronger relationship between objective and subjective function identified within this cohort.

### 4.2 Gait biomechanics and perceived pain

A secondary finding of this study is that changes in biomechanics were not strongly correlated to changes in perceived pain. There was a significant post-operative relationship between B(OA) and PACS (r=-0.623, p = .003) indicating that subjects in more pain following surgery also displayed more pathological biomechanics and vice versa. This relationship wasn’t seen pre-operatively, where there appeared little or no relationship between pain and biomechanics (r=-0.261, p = .328). Within this cohort, therefore, improvements in perceived pain following surgery were not reflective of changes in gait biomechanics.

There is conflicting evidence on the relationship between subjectively assessed pain and objective gait parameters following TKR surgery. Joint pain is known to cause compensatory gait alterations in individuals with knee OA [26], and hence a correlation between these gait biomechanics and pain might be expected pre-operatively. Our finding, that pain is less related to biomechanics before surgery, does not support this hypothesis. Liebensteiner et al. [26] have previously reported a lack of correlation between the Knee Society Pain subscore and objective gait parameters before and after TKR, however, did not include kinetic parameters which may reflect load-avoidance through fear or pain within their analysis. Madeville and colleagues [28] found a moderate correlation between objective measures of gait stability and Western Ontario & McMaster Universities Osteoarthritis Index (WOMAC) pain score before surgery which wasn’t present six months following surgery. Bonnefoy-Mazure and colleagues [29] found no significant relationship between WOMAC pain score and objective gait parameters one year following TKR, however, noted moderate correlations (r = 0.3 - 0.4) between some of these measures and Visual Analogue Scale knee pain.

There is currently a lack of standardisation in the way both patient-perceived pain and objective gait biomechanics are measured following surgery, which may underpin the inconsistency of findings. Pain within the current study was assessed using the PACs score, a generic 11-question score developed by the British Pain Society which later evolved into the Brief Pain Inventory, and has been validated in the assessment of chronic non-malignant pain [30]. The PACS differs from several other outcome measures in that seven of the questions assess the amount that pain has *interfered with* important aspects of life, as opposed to the pain *experienced during* specific movements. The much poorer relationship between pain and biomechanics before surgery is surprising and warrants further investigation. One possible explanation may be a higher use of pain-killers pre-operatively, potentially masking antalgic gait adaptations.

### 4.3 Limitations

There are several limitations to this study which should be acknowledged. Only subjects who could walk 10 m without a walking aid could be included within this study. The results therefore cannot be generalised to all patients undergoing TKR surgery. The data analysed is part of an ongoing study and the statistical power is limited by the relatively small cohort size of the post-operative cohort (n = 22). This is typical of similar biomechanical studies using marker-based motion capture [24,26], and is a consequence of the time-intensive nature of the collection and processing of marker-based motion capture. The NP cohort used to train the classifier was significantly younger and had a lower BMI than the OA subjects. Gait biomechanics are affected by both aging and BMI; recovery of biomechanics must therefore be interpreted as a level of change towards that of a younger, lighter cohort.

Within this study post-operative recovery was considered at a single time-point. There is evidence that objective and subjective measures of function may have different trajectories of improvement, particularly during early recovery [4,24]. The relationship between trajectories of patient-perceived function and objective measures of gait biomechanics warrant further investigation. The utilisation of data reduction and classification techniques introduces the risk of over-fitting, which could over-estimate the accuracy of our ability to discriminate the biomechanical features related to late-stage OA. Steps were taken to reduce the risk of over-fitting (data-splitting and cross-validation). Additionally, the risk of over-fitting is reduced in comparison to common machine-learning techniques as the control parameters of the transfer function are defined explicitly, as opposed to being iteratively optimised.

## 5 Conclusion

Surprisingly strong correlations have been observed in a cohort of 22 TKR subjects between gait biomechanics measures using the Cardiff Classifier and patient perceived measures of function. Current performance-based measures of function which fail to demonstrate a strong correlation with patient-perceived measures warrant further biomechanical investigation to identify whether they adequately reflect changes in the quality of movement.

## Author contributions

P. R Biggs - Acquisition of data, analysis and interpretation of data, drafting the manuscript and revising it critically, final approval of the version to be submitted.

G. M Whatling: Conception and design of the study, acquisition of data, drafting the manuscript and revising it critically, final approval of the version to be submitted.

C. Wilson: Conception and design of the study, TKR surgery for participants in the study, revision of manuscript and clinical interpretation of findings, final approval of the version to be submitted.

C. Holt: Conception and design of the study, revising manuscript critically for important intellectual content, final approval of the version to be submitted.

## Declaration of interest

None.

## References

[1] Losina E., Katz J.N. (2012). Total knee arthroplasty on the rise in younger patients: are we sure that past performance will guarantee future success?. Arthritis Rheum..

[2] Kurtz S.M., Lau E., Ong K., Zhao K., Kelly M., Bozic K.J. (2009). Future young patient demand for primary and revision joint replacement: national projections from 2010 to 2030. Clin. Orthop. Relat. Res..

[3] Nilsdotter A.K., Toksvig-Larsen S., Roos E.M. (2009). Knee arthroplasty: are patients’ expectations fulfilled? A prospective study of pain and function in 102 patients with 5-year follow-up. Acta Orthop..

[4] Mizner R.L., Petterson S.C., Clements K.E., Zeni J.A., Irrgang J.J., Snyder-Mackler L. (2011). Measuring functional improvement after total knee arthroplasty requires both performance-based and patient-report assessments: a longitudinal analysis of outcomes. J. Arthroplasty.

[5] Stratford P.W., Kennedy D.M. (2006). Performance measures were necessary to obtain a complete picture of osteoarthritic patients. J. Clin. Epidemiol..

[6] Jacobs C.A., Christensen C.P. (2009). Correlations between knee society function scores and functional force measures. Clin. Orthop. Relat. Res..

[7] Naili J.E., Iversen M.D., Esbjörnsson A.C., Hedström M., Schwartz M.H., Häger C.K., Broström E.W. (2017). Deficits in functional performance and gait one year after total knee arthroplasty despite improved self-reported function, Knee Surgery, Sport. Traumatol. Arthrosc..

[8] McClelland J.A., Webster K.E., Feller J.A. (2007). Gait analysis of patients following total knee replacement: a systematic review. Knee..

[9] Schwartz M.H., Rozumalski A. (2008). The Gait Deviation Index: a new comprehensive index of gait pathology. Gait Posture.

[10] Schutte L.M., Narayanan U., Stout J.L., Selber P., Gage J.R., Schwartz M.H. (2000). An index for quantifying deviations from normal gait. Gait Posture.

[11] Cimolin V., Galli M. (2014). Summary measures for clinical gait analysis: a literature review. Gait Posture.

[12] Deluzio K.J., Wyss U.P., Costigan P.A., Sorbie C., Zee B. (1999). Gait assessment in unicompartmental knee arthroplasty patients: principal component modelling of gait waveforms and clinical status. Hum. Mov. Sci..

[13] Astephen J.L., Deluzio K.J., Caldwell G.E., Dunbar M.J. (2008). Biomechanical changes at the hip, knee, and ankle joints during gait are associated with knee osteoarthritis severity. J. Orthop. Res..

[14] Beynon M.J., Jones L., Holt C.A. (2006). Classification of osteoarthritic and normal knee function using three-dimensional motion analysis and the Dempster-Shafer theory of evidence. Syst. Man Cybern. Part A Syst. Humans IEEE Trans..

[15] Jones L., Beynon M.J., Holt C.A., Roy S. (2006). An application of the Dempster–Shafer theory of evidence to the classification of knee function and detection of improvement due to total knee replacement surgery. J. Biomech..

[16] Worsley P.R., Whatling G., Barrett D., Holt C., Stokes M., Taylor M. (2016). Assessing changes in subjective and objective function from pre-to post-knee arthroplasty using the Cardiff Dempster–Shafer theory classifier. Comput. Methods Biomech. Biomed. Engin..

[17] Metcalfe A.J., Stewart C.J., Postans N.J., Biggs P.R., Whatling G.M., Holt C.A., Roberts A.P. (2017). Abnormal Loading and Functional Deficits Are Present in Both Limbs Before and After Unilateral Knee Arthroplasty.

[18] Cappozzo A., Catani F., Della Croce U., Leardini A. (1995). Position and orientation in space of bones during movement: anatomical frame definition and determination. Clin. Biomech..

[19] Sapatinas T. (2004). The Elements of Statistical Learning.

[20] Cohen J. (1988). Statistical Power Analysis for the Behavioral Sciences.

[21] World Health Organization (2001). International Classification of Functioning, Disability and Health : ICF. https://www.who.int/classifications/icf/en/.

[22] Graff C., Hohmann E., Bryant A.L., Tetsworth K. (2016). Subjective and objective outcome measures after total knee replacement: is there a correlation?. ANZ J. Surg..

[23] Ko V., Naylor J.M., Harris I.A., Crosbie J., Yeo A.E. (2013). The six-minute walk test is an excellent predictor of functional ambulation after total knee arthroplasty. BMC Musculoskelet. Disord..

[24] Senden R., Grimm B., Meijer K., Savelberg H., Heyligers I.C. (2011). The importance to including objective functional outcomes in the clinical follow up of total knee arthroplasty patients. Knee.

[25] Dobson F., Hinman R.S., Hall M., Terwee C.B., Roos E.M., Bennell K.L. (2012). Measurement properties of performance-based measures to assess physical function in hip and knee osteoarthritis: a systematic review. Osteoarthr. Cartil..

[26] Liebensteiner M.C., Herten A., Gstoettner M., Thaler M., Krismer M., Bach C.M. (2008). Correlation between objective gait parameters and subjective score measurements before and after total knee arthroplasty. Knee.

[27] Naili J.E., Esbjörnsson A.C., Iversen M.D., Schwartz M.H., Hedström M., Häger C.K., Broström E.W. (2017). The impact of symptomatic knee osteoarthritis on overall gait pattern deviations and its association with performance-based measures and patient-reported outcomes. Knee.

[28] Mandeville D., Osternig L.R., Chou L.-S. (2008). The effect of total knee replacement surgery on gait stability. Gait Posture.

[29] Bonnefoy-Mazure A., Armand S., Sagawa Y., Suvà D., Miozzari H., Turcot K., Kinematic Knee (2017). Clinical outcomes evolution before, 3 months, and 1 year after total knee arthroplasty. J. Arthroplasty.

[30] Tan G., Jensen M.P., Thornby J.I., Shanti B.F. (2004). Validation of the brief pain inventory for chronic nonmalignant pain. J. Pain.

